# Identification and Validation of a Potential Prognostic 7-lncRNA Signature for Predicting Survival in Patients with Multiple Myeloma

**DOI:** 10.1155/2020/3813546

**Published:** 2020-11-05

**Authors:** Yun Zhong, Zhe Liu, Dangchi Li, Qinyuan Liao, Jingao Li

**Affiliations:** ^1^Department of Lymphohematology and Oncology, Jiangxi Cancer Hospital of Nanchang University, Nanchang, Jiangxi, China; ^2^Department of Orthopedics, Jiangxi Cancer Hospital of Nanchang University, Nanchang, Jiangxi, China; ^3^Jiangxi University of Technology High School, Nanchang, Jiangxi, China; ^4^Department of Immunology, Guilin Medical University, Guilin, Guangxi, China; ^5^Department of Radiation Oncology, Jiangxi Cancer Hospital of Nanchang University, Nanchang, Jiangxi, China

## Abstract

**Background:**

An increasing number of studies have indicated that the abnormal expression of certain long noncoding RNAs (lncRNAs) is linked to the overall survival (OS) of patients with myeloma.

**Methods:**

Gene expression data of myeloma patients were downloaded from the Gene Expression Omnibus (GEO) database (GSE4581 and GSE57317). Cox regression analysis, Kaplan-Meier, and receiver operating characteristic (ROC) analysis were performed to construct and validate the prediction model. Single sample gene set enrichment (ssGSEA) and Kyoto Encyclopedia of Genes and Genomes (KEGG) analysis were used to predict the function of a specified lncRNA.

**Results:**

In this study, a seven-lncRNA signature was identified and used to construct a risk score system for myeloma prognosis. This system was used to stratify patients with different survival rates in the training set into high-risk and low-risk groups. Test set, the entire test set, the external validation set, and the myeloma subtype achieved the authentication of the results. In addition, functional enrichment analysis indicated that 7 prognostic lncRNAs may be involved in the tumorigenesis of myeloma through cancer-related pathways and biological processes. The results of the immune score showed that IF_I was negatively correlated with the risk score. Compared with the published gene signature, the 7-lncRNA model has a higher C-index (above 0.8).

**Conclusion:**

In summary, our data provide evidence that seven lncRNAs could be used as independent biomarkers to predict the prognosis of myeloma, which also indicated that these 7 lncRNAs may be involved in the progression of myeloma.

## 1. Introduction

Multiple myeloma (MM) is a blood malignant tumor caused by abnormal proliferation of plasma cells, which is mainly characterized by abnormal proliferation and accumulation of multifocal clonal plasma cells in bone marrow, and the production of a large number of monoclonal immunoglobulin (Ig G, Ig A, Ig D, or Ig E) or its fragments (M protein) [1]. At present, the global incidence of MM is about 4/100,000, and the incidence of MM in China is 2-3/100,000, which has surpassed acute leukemia and become the second most common hematologic malignancy; the incidence is still on the rise in China and the world [2]; this may be related to the deterioration of environmental factors, the aging of the population, and the improvement of a diagnostic level.

Long noncoding RNA (lncRNA) is a type of RNA with more than 200 nucleotides and cannot synthesize proteins [3]. These lncRNAs are involved in posttranscriptional regulation and are abnormally expressed in several solid tumors and hematopoietic malignancies [4–6]. Abnormal expression of several lncRNAs in MM has been reported, and their clinical significance, biological function, and potential molecular mechanism have also been reported [7]. Immunoregulatory drugs (such as lenalidomide and pomamide), proteasome inhibitors (such as bortezomib and carfilzomib), and monoclonal antibodies have significantly improved survival in MM patients over the past decade. However, the treatment of recurrent and some refractory patients remains challenging. Part of the reason is that the pathogenesis and progression of MM involve complex and heterogeneous genomic changes that are significant, including the effects of lncRNA [8].

Some lncRNAs play an important role in the progression of MM and can be used as a prognostic indicator in MM patients. For example, metastatic-associated lung adenocarcinoma transcript 1 (MALAT1) is overexpressed in MM tissues and various MM cell lines, and upregulation of MALAT1 is significantly associated with poor prognosis in MM [8, 9]. Nuclear paraspeckle assembly transcript (NEAT1) also plays a key role in promoting MM, and its increased expression is closely related to poor prognosis [10]. Colon cancer-associated transcript 1 (CCAT1) is closely associated with poor MM prognosis [11]. Despite the large number of members of the lncRNA family, only a few are associated with the prognosis of MM. In addition, the predictive power of a single indicator is limited, so a prognostic model composed of multiple indicators is needed in clinical practice for the comprehensive clinical evaluation of tumor prognosis. Prognostic models combining several prognostic indicators have been used in a variety of other tumors [12, 13]. However, MM prognostic model with multiple lncRNAs has not been reported.

Therefore, in this study, gene microarray with MM prognostic data was firstly screened; lncRNA closely related to the prognosis was statistically analyzed to construct the MM prognostic model. We applied the prognostic model to multiple datasets and molecular subtypes to confirm the prognostic performance of the model and to compare it with published models. The prognostic model of MM is expected to provide a new direction for the clinical application of MM.

## 2. Material and Methods

### 2.1. Data Source

**Figure 1:**
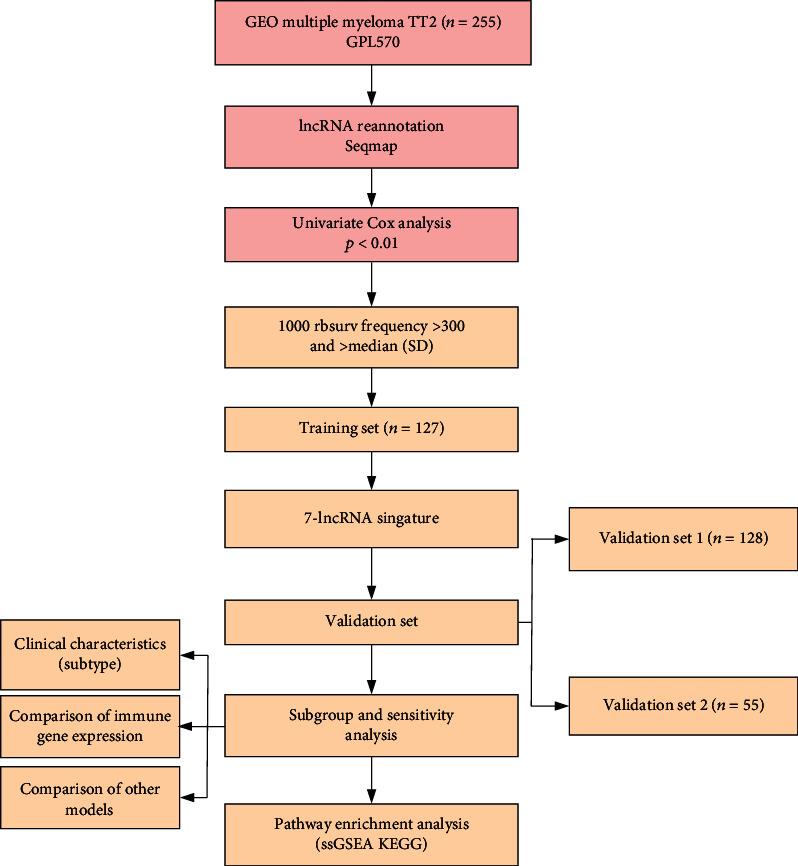
Work chart.

Based on the Affymetrix-GPL570 platform, the expression of the probe, gene expression data, and samples of follow-up information were derived from the national center for biotechnology information Gene Expression Omnibus (GEO) (http://www.ncbi.nlm.nih.gov/geo/). Datasets GSE4581 (*n* = 256) and GSE57317 (*n* = 55) were used for myeloma network analysis. Among them, the GSE4581 is randomly divided into a training set (127 samples) and an internal validation set (128 samples) according to the ratio of 1 : 1. The GSE57317 as an external validation set contains 55 samples. The specific distribution of survival state, median survival time, and molecular subtypes in the two sets of datasets is shown in Table 1. The work flow chart is shown in Figure 1.

### 2.2. lncRNA Reannotated

lncRNAs were annotated using a large number of probes from the Affymetrix HG-U133 Plus 2.0 microarray. Briefly, Affymetrix probe sequences were downloaded from the website (http://www.affymetrix.com) and mapped precisely to the human genome (hg38) via Bowtie. The chromosomal positions of the probes were matched to the chromosomal positions of the lncRNAs to obtain lncRNA-specific probes according to the annotation of GENCODE (release 32) [14]. By using BEDTools (http://code.google.com/p/bedtools) [15], we selected probes that fell completely into the lncRNA exon without overlapping with protein-coding genes. The expression value of one lncRNA gene detected by at least 5 probes was kept. The expression level of the lncRNA is indicated by the median expression value of multiple probes mapped to the same lncRNA. The expression data from each cohort were log2 transformed and normalized using a quantile normalization method. Finally, two corresponding lncRNA expression datasets were constructed, containing 4094 lncRNAs.

### 2.3. Univariate Cox Survival Analysis

Univariate Cox analysis was performed using the R package survival coxph function [16] to select prognostic lncRNA. *p* < 0.05 was considered statistically significant. Prognostic lncRNAs are divided into protective factors and risk factors.

### 2.4. Prognostic Survival Model

Based on these prognostic lncRNAs, the best prognostic lncRNA group was selected by using a robust likelihood-based survival model using the R package rbsurv [17]. The software package selects survival-related genes by separating two groups of survival-related genes as a cross-validation technique with large variability. It uses forward selection to generate a number of gene models and selects the optimal model according to the Abscissa Information Criteria (AIC). Briefly, 75% of all samples in the training set were randomly selected using threefold cross-validation. The maximum number of genes was selected to be 30, and the analysis was repeated 1000 times. Then, the selected key lncRNAs were included in multivariate Cox analysis, and a risk score formula was constructed:

(1) $$\begin{array}{c} \text{Risk score}=\overset{n}{\underset{k=1}{\sum }}{\text{Exp}}_{k}*{e^{\text{HR}}}_{k}, \end{array}$$

where *N* is the number of prognostic lncRNAs, Exp_*k*_ is the expression value of prognostic lncRNAs, and *e*^HR^_*k*_ is the estimated regression coefficient of lncRNAs in the multivariate Cox regression analysis.

### 2.5. ROC Curve Construction

The performance of prognostic models was tested using the timeROC package in the R software [18], which calculates receiver-operator characteristic (ROC) curves [19]. Area under the curve (AUC) was plotted to evaluate the prognostic value.

### 2.6. Functional Enrichment Analysis

Gene Set Enrichment Analysis (GSEA) was proposed in 2005 to analyze the expression of a group of functionally related genes based on gene expression profile data [20]. ssGSEA (single sample gene enrichment analysis) is an extension of the GSEA algorithm. The ssGSVA algorithm is implemented by the R software package GSVA [21]. Gene sets with a *p* value less than 0.05 after performing 1000 permutations were considered to be significantly enriched.

### 2.7. Immune Score

According to the method published by Safonov et al. [22], the scores of 13 immune factors were calculated and the differences of immune factor scores in the high/low samples of the training set were compared. The correlation between the significantly different immune factors and risk score was further compared.

### 2.8. Comparison with Published Models

We select 2 published related risk models, one of which is a 16-gene signature [23] and the other is a 6-gene signature [24], which was compared with our 7-lncRNA signature. The ROC and Kaplan-Meier (KM) survival curves of the published models in the training set and the C-index of the three models are plotted to compare the optimal models.

### 2.9. Statistical Analysis

The KM curve was plotted when the median risk score in each dataset was used as a cutoff to compare the risk of survival between the high-risk group and the low-risk group. Multivariate Cox regression analysis was performed to test whether lncRNA markers were independent prognostic factors. Significance was defined as *p* < 0.05. A heat map was drawn using the R package pheatmap. All analyses used default parameters except for special instructions, which are performed in the R software version 3.4.3.

## 3. Results

### 3.1. Identification of lncRNA with a Significant Prognosis in Myeloma

First, we performed a univariate Cox proportional hazards regression model on 4094 reannotated lncRNA expression levels and survival data in the training set samples using the R package survival coxph function, *p* < 0.01 as the threshold. We finally obtained 72 probes with a significant prognosis, of which the most significant top 20 lncRNAs are shown in Table 2.

### 3.2. Identification of a 7-lncRNA Signature Risk Model and Survival Analysis

**Figure 2:**
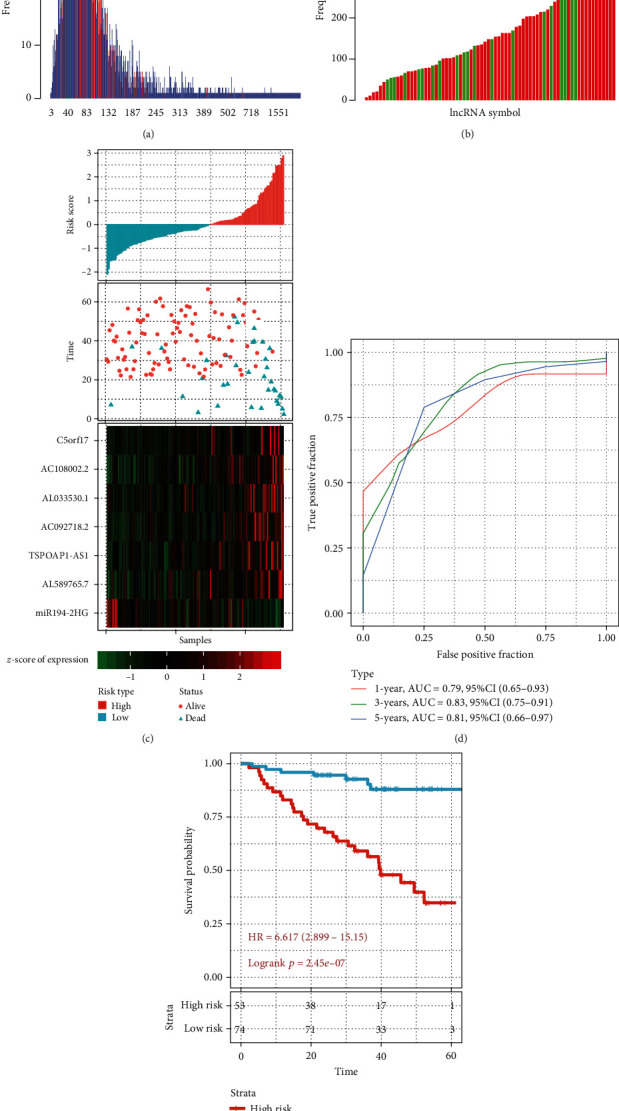
Identification of a 7-lncRNA signature risk model and survival analysis. (a) The distribution of the standard deviation of lncRNAs: red indicates the position of the standard deviation of lncRNA probes whose frequency is greater than 300, the horizontal axis indicates the standard deviation, and the vertical axis indicates the number of probes. (b) The frequency distribution of lncRNA selected by the rbsurv feature a thousand times. The horizontal axis represents lncRNA, and the vertical axis represents the frequency of occurrence. Red indicates that the standard deviation of the lncRNA probe is greater than the median of the overall standard deviation, and green indicates that it is less than the median of the overall standard deviation. (c) Risk score, survival time, survival status, and expression of 7 lncRNAs in the training set. (d) ROC curve and AUC of a 7-lncRNA signature. (e) KM survival curve distribution of a 7-lncRNA signature in the training set.

Prognostic lncRNAs were further selected; rbsurv analysis was performed on 75% samples randomly selected from the training set samples. The frequency of each probe in the 1000 rbsurv analysis showed that the frequency of most probes was around 10%, suggesting that the influence of these probes on prognosis was not stable in different sample sets (Figure 2(a)). The standard deviation of these lncRNA probes was calculated. lncRNAs with a standard deviation greater than the median standard deviation of all probes and frequency greater than 300 were selected, including 11 lncRNAs (Figure 2(b)). First, KM curve analysis showed that only AC092718.4, AC093673.1, and AC234582.2 could not be divided into two groups with high and low risk, and only the group with low expression of miR194-2HG had poor prognosis (Figure S1). Eight lncRNAs with *p* < 0.05 in the KM curve were analyzed by multivariate Cox survival analysis, and the 7 lncRNAs with the lowest AIC value (AIC = 266.62) were retained as the final model (Table 3). The model is shown as follows: risk score = 0.002∗AC092718.2 + 0.005∗AC108002.2 + 0.001∗AL033530.1 + 0002∗AL589765.7 + 0.003∗C5orf17‐0.004∗miR194‐2HG + 0.003∗TSPOAP1‐AS1.

According to the expression levels of 7 lncRNAs in the training set samples, the risk scores of each sample were calculated, respectively, and the risk score of the samples was plotted. The survival samples showed that the number of deaths of samples with high-risk scores was significantly higher than those with low-risk scores. The expression of 7 lncRNAs in the samples showed that the high expression of C5orf17, AC092718.2, AC108002.2, AL033530.1, AL589765.7, and TSPOAP1-AS was associated with high risk, which was a risk factor; the high expression of miR194-2HG was associated with low risk, which was a protective factor (Figure 2(c)). Further, we used the R software package timeROC to conduct ROC analysis on risk score and analyzed the prognostic classification efficiency of 1 year, 3 years, and 5 years, respectively. The results showed that the model had a high AUC line area and AUC > 0.79 (Figure 2(d)). Next, we conducted *z*-score on risk score and divided the samples with risk score greater than zero into the high-risk group, with a total of 53 samples, and those with a risk score less than zero into the low-risk group, with a total of 74 samples. The KM prognostic survival curve indicated that there was a significant difference between the two groups (Logrank *p* < 0.0001, HR = 6.617 (2.899-15.15) (Figure 2(e))).

### 3.3. Robustness of a 7-lncRNA Signature

**Figure 3:**
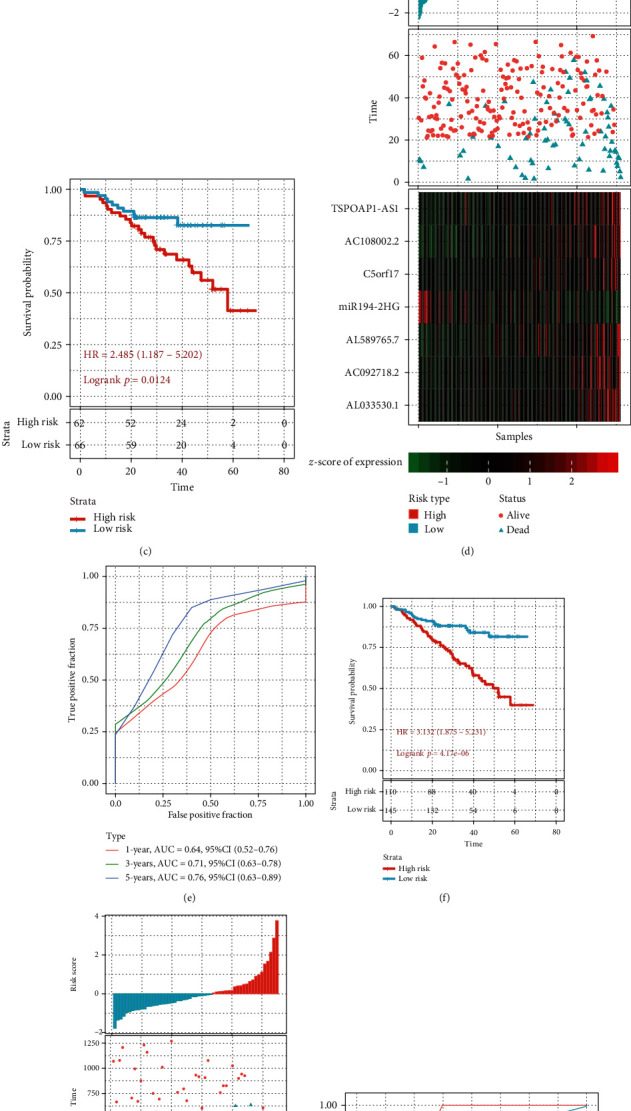
The robustness of the 7-lncRNA signature. (a) Risk score, survival time, survival status, and expression of 7 lncRNA in the internal validation set GSE4581. (b) ROC curve and AUC of a 7-lncRNA signature in the internal validation set GSE4581. (c) KM survival curve distribution of a 7-lncRNA signature in the internal validation set GSE4581. (d) Risk score, survival time, survival status, and expression of 7 lncRNAs in the entire dataset GSE4581. (e) ROC curve and AUC of a 7-lncRNA signature in the entire dataset GSE4581. (f) KM survival curve distribution of a 7-lncRNA signature in the entire dataset GSE4581. (g) Risk score, survival time, survival status, and expression of 7-lncRNA in the external validation set GSE57317. (h) ROC curve and AUC of a 7-lncRNA signature in the external validation set GSE57317. (i) KM survival curve distribution of a 7-lncRNA signature in the external validation set GSE57317.

In order to determine the robustness of the model, we use the same model and the same coefficients as the training set in the internal validation set GSE4581, the entire dataset GSE4581, and the external validation set GSE57317. We also calculate the risk score of each sample according to the expression level of the sample and draw the risk score distribution of the sample. The OS with the higher risk score is significantly smaller than the one with the lower risk score. Similarly, high expressions of C5orf17, AC092718.2, AC108002.2, AL033530.1, AL589765.7, and TSPOAP1-AS were associated with high risk, as a risk factor. High expression of miR194-2HG is related to low risk as a protective factor (Figures 3(a), 3(d), and 3(g)). ROC curve analysis shows that the five-year AUC is higher than 0.7 (Figures 3(b), 3(e), and 3(h)). Finally, the KM prognosis analysis was performed, and the data showed that there were significant differences between high- and low-risk groups (Figures 3(c), 3(f), and 3(i)). These results indicate that the 7-lncRNA signature has good robustness.

### 3.4. Identification of the Relationship between Risk Score and Function

**Figure 4:**
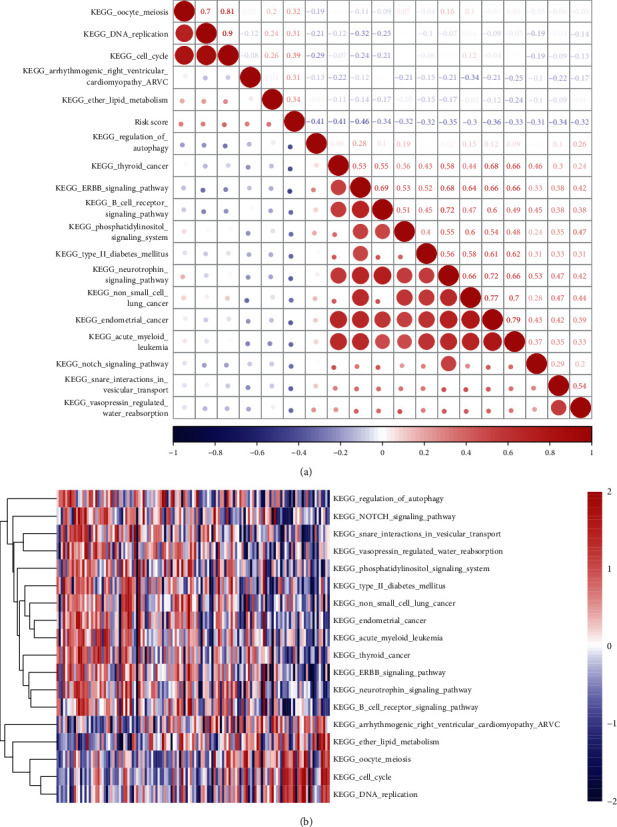
Relationship between sample risk score and biological function. (a) Clustering of correlation coefficients between KEGG pathways with a correlation with risk score greater than 0.3 and risk score. (b) The changes in the relationship of the KEGG pathway in ssGSEA score in each sample with the increase of risk score. The horizontal axis represents the sample, and the risk score increases successively from left to right.

The R software package GSVA was used to calculate the score of each sample on different functions, the correlation between these functions and the risk score was calculated, and the function with a correlation greater than 0.3 was selected. The results showed that most of the functions were negatively correlated with the risk score of the sample, and a few were positively correlated with the risk score (Figure 4(a)). 18 KEGG pathways, with a correlation greater than 0.3, were selected for clustering analysis; it is obvious that among these 18 pathways, DNA replication, cell cycle, ether lipid metabolism, etc. increase with the rise of the risk score and non-small-cell lung cancer, thyroid cancer, NOTCH signaling pathway, and other related pathways decrease with increasing risk scores, which also suggests that the imbalance of these pathways may be closely related to the development of myeloma (Figure 4(b)).

### 3.5. Correlation between a Risk Model and an Immune Score

**Figure 5:**
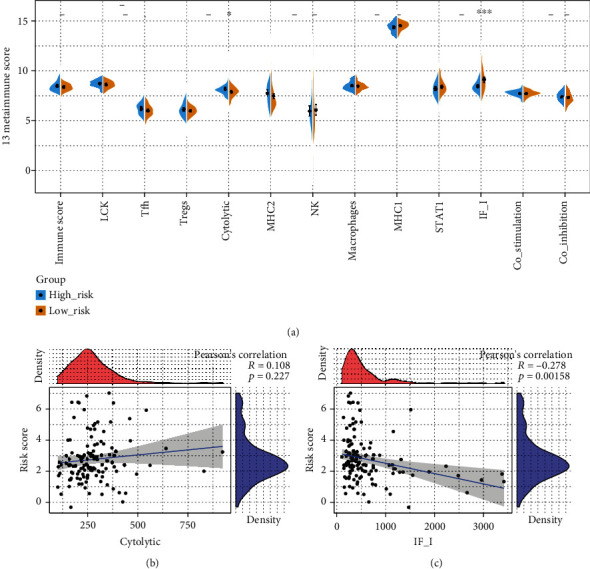
Correlation analysis between a risk model and an immune score. (a) Differences of 13 immune factor scores in high- and low-risk groups. (b) The correlation between Cytolytic and risk score. (c) Correlation between IF_I and risk score, where the red density plot represents the distribution of immune scores, and the blue density plot represents the distribution of risk score.

In order to identify the relationship between the risk score of a 7-lncRNA signature and the immune score, the scores of 13 immune factors were first calculated. The significant difference between the immune factor scores in the high/low-risk samples of the training set shows that only IF_I and Cytolytic show significant differences in the high- and low-risk groups, *p* < 0.05 (Figure 5(a)). Next, we calculated the correlation between the two significant immune scores and the risk score and found that IF_I showed a significantly negative correlation with the risk score (Figure 5(c)). Although Cytolytic is positively correlated with the risk score correlation trend, it is not significant (Figure 5(b)). It shows that our risk model may have some connection with IF_I.

### 3.6. Relationship between a Risk Model and a Molecular Subtype

**Figure 6:**
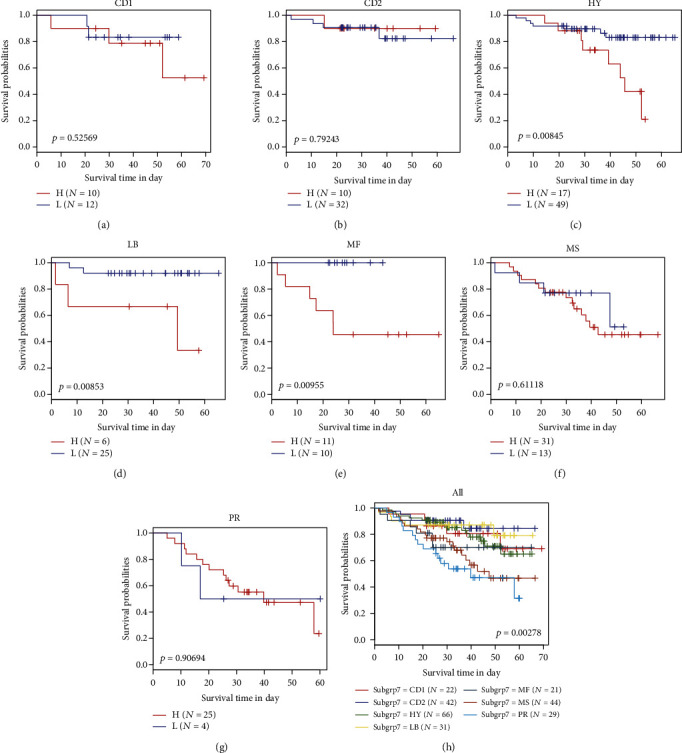
The risk model showed significant differences in the prognosis of the seven subtypes. (a–h) KM survival curves of 7 subtypes, in which blue represents the low-expression group and red represents the high-expression group.

In order to analyze the predictive efficacy of a 7-lncRNA signature in different subtypes, our model could significantly divide hyperdiploid, low bone disease, and MAF/MAFB subtypes into two groups with high and low risk, and the prognosis is significantly different (Figures 6(a)–6(g)). The analysis showed that there were significant differences in the prognosis of 7 subtypes (Figure 6(h)).

### 3.7. Comparison of Risk Models with Other Models

**Figure 7:**
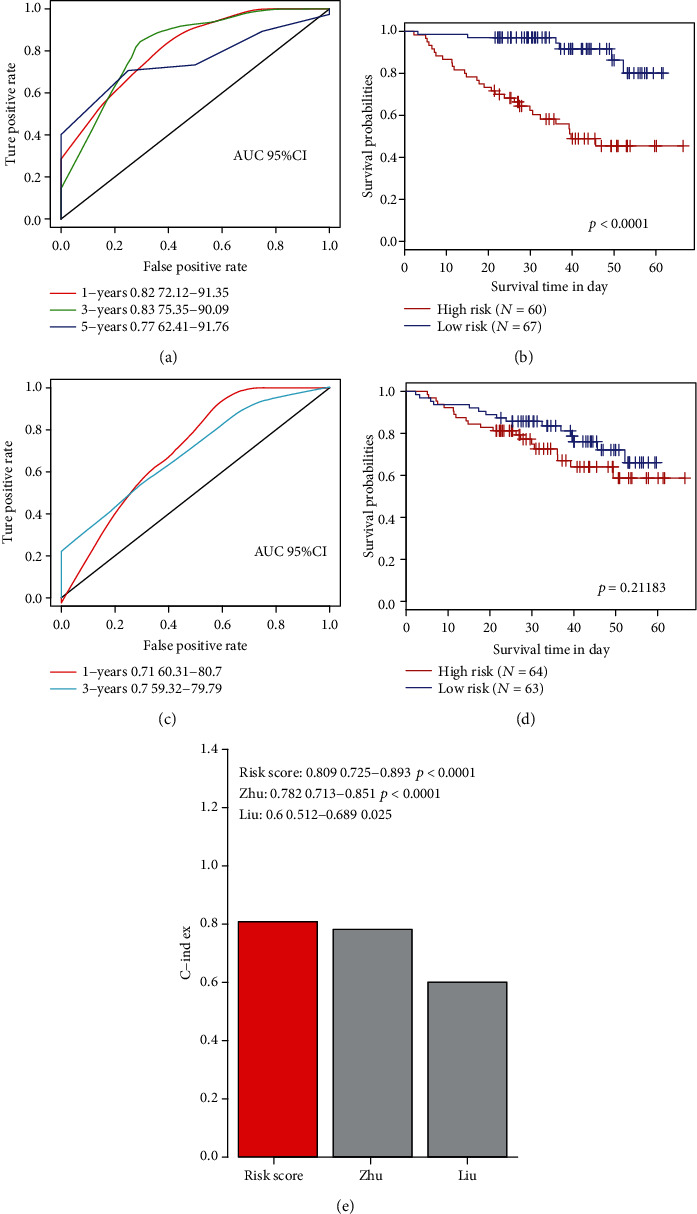
Comparison of risk models with other models. (a) AUC curve of a 16-gene signature in the training set. (b) Prognostic KM curve of a 16-gene signature in the training set. (c) AUC curve of a 6-gene signature in the training set. (d) Prognostic KM curve of a 6-gene signature in the training set. (e) Comparison of C-index in three models.

Two published risk models were selected, one of which was a 16-gene signature [23] and the other was a 6-gene signature [24], compared with our 7-lncRNA signature. In order to make the model comparable, we use multifactor Cox analysis to calculate the risk score of the training set samples based on the corresponding genes in the model, evaluate the ROC of the two models, and divide the samples into high according to the optimal threshold. The risk prognosis of the two groups of samples was calculated for the low-risk and high-risk groups. The 16-gene signature ROC and KM curve results showed that the 3-year AUC was 0.83 (Figure 7(a)), and the prognosis was significantly different (*p* < 0.0001) (Figure 7(b)). ROC and KM curve of 6-gene signature results showed that the 1-year AUC value was 0.71 (Figure 7(c)), but the prognosis was not significant (Figure 7(d)). In order to compare the predictive performance of these models on myeloma samples, we use the rms package in R to calculate the concordance index (C-index) of our 2 models and our model. The C-index of the 7-lncRNA model in the 3 models is above 0.8 (Figure 7(e)); the overall performance of the 7-lncRNA signature model is better than the other two.

## 4. Discussion

In the past period of time, great progress has been made in the understanding of the occurrence and development of MM [25]. However, the clinical characteristics of myeloma patients remain highly heterogeneous. The traditional laboratory parameters S, 2M, and serum albumin, known as the international staging system (ISS), have been used as an objective staging system [26]. Cytogenetic studies have found cytogenetic abnormalities, such as 13q14 deletions and t (4; 14) translocation that can also provide valuable prognostic information [27, 28]. With the development of high-throughput techniques, molecular markers based on expression profiles have been reported in various types of cancer, and these markers have become more effective prognostic tools for predicting the prognosis of patients [29]. A number of multigene expression features have been developed, including the UAMS 17 gene [30] and the IFM 15 gene model [31], which have been developed to predict survival in MM patients. Recently, dysregulation of lncRNA expression has been observed in newly diagnosed MM patients, indicating their potential as biomarkers for the diagnosis and prognosis of MM [32]. However, there are few reports on the prognostic significance of lncRNA signature based on the expression profile for the prognosis of MM patients.

Zhou et al. analyzed the data of the GSE24080 gene chip and randomly divided MM patients into a training dataset (*n* = 280) and test dataset (*n* = 279) [33]. The team used a univariate regression analysis to find 59 lncRNAs that were closely related to patient OS. After multivariate regression analysis, four lncRNAs (RP4-803J11.2, RP1-43E13.2, RP11-553L6.5, and ZFY-AS1) were shown to have predictive effects. Hu et al. identified 176 lncRNAs significantly related to the survival status of MM patients from the GSE24080 and GSE57317 datasets, especially RP1-286D6.1, AC008875.2, MTMR9L, AC069360.2, and AL512791.1, which can be used to evaluate the prognosis of MM patients [34]. None of the lncRNAs found in the two studies above overlapped with the lncRNAs associated with the top 20 prognosis found in this study. The different results of their study and this study may lie in the use of different statistical tools and different GEO databases. Hu et al. did not conduct ROC analysis to check the prognostic value of lncRNA, while Zhou et al. found that the AUC of four lncRNA signature had 0.682. In this study, we found that the AUC of the lncRNA prognostic model was greater than 0.79 (>0.682), higher than that of Zhou et al.'s ROC, which had certain advantages in predicting the survival status of MM patients.

lncRNA in the prognostic model constructed in this study has not been studied in MM. Only three lncRNAs (C5orf17, miR194-2HG, and TSPOAP1-AS1) have been more or less studied in different diseases. Qi et al. constructed five lncRNA prognostic models for lung squamous cell carcinoma including C5orf17 [35]. The ceRNA network indicates that lncRNA may contain bladder cancer-related microRNA (miRNA) recognition elements [36]. The expression of host lncRNA TSPOAP1-AS1 was significantly induced by influenza A virus (IAV) infection [37]. Giulietti et al. performed a survival analysis and identified TSPOAP1-AS1 as prognostic biomarkers for pancreatic cancer [38].

Inevitably, there are some shortcomings in the research work, which we hope to solve in the future work. First, although 256 cases were included in this study, our findings should be confirmed in a separate cohort. Second, the prognostic value of lncRNA was studied using gene microarray. This single detection method should also be verified by other methods, such as real-time RT-qPCR. Third, most of the lncRNA in our prognostic model has not been reported. Their specific clinical significance, biological function, and potential mechanism of action should be studied in further experiments. In summary, more experimental evidence is needed to determine how prognostic lncRNA functions in MM.

## 5. Conclusion

In this study, we developed the prognostic marker of myeloma OS (7-lncRNA signature) through bioinformatics methods, which may contribute to the understanding of the disorder RNA involved in the development and prognosis of myeloma and will lay the foundation for the development of novel clinical diagnostic and therapeutic biomarkers.

## Figures and Tables

**Table 1:** Clinical information of two datasets.

Characteristic		Training datasets (*n* = 127)	Validation datasets (*n* = 128)	*p*	GSE57317 (*n* = 55)
Survival status	Living	92	94	0.969	43
	Dead	35	34		12
Survival time	High risk	31.73	26.02		17
	Low risk	37.53	34.69		23.47
Mol. subtype	CD-1: CCND1/CCND3 (group 5)	11	11	0.882	5
	CD-2: CCND1/CCND3 (group 6)	21	21		13
	HY: hyperdiploid	28	38		13
	LB: low bone disease	16	15		6
	MF: MAF/MAFB	12	9		2
	MS: MMSET	23	21		3
	PR: proliferation	16	13		13

**Table 2:** Top 20 most significant lncRNA probes.

Symbol	*p* value	HR	Low 95% CI	High 95% CI
AL033530.1	8.11*E* − 06	1.002	1.001	1.003
TSPOAP1-AS1	1.16*E* − 05	1.005	1.003	1.008
AC108002.2	2.74*E* − 05	1.005	1.003	1.007
AC109322.1	4.53*E* − 05	1.003	1.001	1.004
AC092718.2	5.02*E* − 05	1.003	1.001	1.004
AL589765.7	9.32*E* − 05	1.000	1.000	1.001
AL078590.2	0.000136561	0.993	0.990	0.997
C5orf17	0.000149994	1.004	1.002	1.005
AP004608.1	0.000342609	1.004	1.002	1.006
AC092718.4	0.000345437	1.003	1.001	1.004
AC067852.2	0.000546518	0.996	0.994	0.998
AC022784.3	0.000777743	1.012	1.005	1.019
AC108488.1	0.000816299	1.005	1.002	1.009
AC234582.2	0.000935172	1.001	1.000	1.001
CRNDE	0.001129051	1.000	1.000	1.001
AC025048.4	0.001531354	1.003	1.001	1.004
AP000777.3	0.001565427	1.000	1.000	1.001
AC096734.2	0.001779434	1.003	1.001	1.005
MIR503HG	0.001782226	1.004	1.002	1.007
LINC00507	0.001784471	1.015	1.005	1.024

**Table 3:** Seven lncRNAs.

lncRNAs	*p*	HR	*z*	Low 95% CI	High 95% CI
AC092718.2	0.04719	1.0016	1.9850	1.0000	1.0031
AC108002.2	0.000244	1.0045	3.6690	1.0021	1.0069
AL033530.1	0.057083	1.0011	1.9030	1.0000	1.0022
AL589765.7	0.018056	1.0003	2.3640	1.0001	1.0006
C5orf17	0.001176	1.0033	3.2450	1.0013	1.0052
miR194-2HG	0.032055	0.9962	-2.1440	0.9927	0.9997
TSPOAP1-AS1	0.06837	1.0026	1.8230	0.9998	1.0054

## Data Availability

The analyzed datasets generated during the study are available from the corresponding author on reasonable request. The RNA-Seq data have been deposited at the Gene Expression Omnibus (see URLs) under the accession number GSE4581 and GSE57317.

## Supplementary Materials

Supplementary Materials KM curve analysis of 11 lncRNAs.

## References

[1] Mayani H. (2013). Hematopoietic and microenvironment alterations in bone marrow from patients with multiple myeloma. *Leukemia Research*.

[2] Rajkumar S. V. (2016). Multiple Myeloma: 2016 Update on diagnosis, risk-stratification, and management. *American journal of hematology*.

[3] Wu S., Bono J., Tao Y. X. (2019). Long noncoding Rna (Lncrna): a target in neuropathic pain. *Expert Opinion on Therapeutic Targets*.

[4] Chen Y. K., Yen Y. (2019). The ambivalent role of Lncrna Xist in carcinogenesis. *Stem Cell Reviews and Reports*.

[5] Xue J. Y., Huang C., Wang W., Li H. B., Sun M., Xie M. (2018). Hoxa 11-As: a novel regulator in human cancer proliferation and metastasis. *Oncotargets and Therapy*.

[6] Huang H., Sun J., Sun Y. (2019). Long noncoding Rnas and their epigenetic function in hematological diseases. *Hematological Oncology*.

[7] Butova R., Vychytilova-Faltejskova P., Souckova A., Sevcikova S., Hajek R. (2019). Long non-coding Rnas in multiple myeloma. *Non-coding RNA*.

[8] Willenbacher W., Seeber A., Steiner N. (2018). Towards molecular profiling in multiple myeloma: a literature review and early indications of its efficacy for informing treatment strategies. *International Journal of Molecular Sciences*.

[9] Ronchetti D., Agnelli L., Taiana E. (2016). Distinct Lncrna transcriptional fingerprints characterize progressive stages of multiple myeloma. *Oncotarget*.

[10] Wu Y., Wang H. (2018). Lncrna Neat1 promotes dexamethasone resistance in multiple myeloma by targeting Mir-193a/Mcl1 pathway. *Journal of Biochemical and Molecular Toxicology*.

[11] Chen L., Hu N., Wang C., Zhao H., Gu Y. (2018). Long non-coding Rna Ccat1 promotes multiple myeloma progression by acting as a molecular sponge of Mir-181a-5p to modulate Hoxa1 expression. *Cell Cycle*.

[12] Kim H. Y., Lee D. H., Lee J. H. (2018). Novel biomarker-based model for the prediction of sorafenib response and overall survival in advanced hepatocellular carcinoma: a prospective cohort study. *BMC Cancer*.

[13] Zhou M., Zhang Z., Zhao H., Bao S., Sun J. (2018). A novel Lncrna-focus expression signature for survival prediction in endometrial carcinoma. *BMC Cancer*.

[14] Harrow J., Denoeud F., Frankish A. (2006). Gencode: producing a reference annotation for encode. *Genome biology*.

[15] Quinlan A. R., Hall I. M. (2010). Bedtools: a flexible suite of utilities for comparing genomic features. *Bioinformatics*.

[16] Emura T., Matsui S., Chen H. Y. (2019). Compound.Cox: univariate feature selection and compound covariate for predicting survival. *Computer Methods and Programs in Biomedicine*.

[17] Moni M. A., Liò P. (2014). Comor: a software for disease comorbidity risk assessment. *Journal of clinical bioinformatics*.

[18] Hoo Z. H., Candlish J., Teare D. (2017). What is an roc curve?. *Emergency Medicine Journal*.

[19] Lemos T., Kalivas J. H. (2020). Self-optimized one-class classification using sum of ranking differences combined with a receiver operator characteristic curve. *Analytical Chemistry*.

[20] Subramanian A., Tamayo P., Mootha V. K. (2005). Gene set enrichment analysis: a knowledge-based approach for interpreting genome-wide expression profiles. *Proceedings of the National Academy of Sciences of the United States of America*.

[21] Hänzelmann S., Castelo R., Guinney J. (2013). Gsva: gene set variation analysis for microarray and Rna-Seq data. *BMC Bioinformatics*.

[22] Safonov A., Jiang T., Bianchini G. (2017). Immune gene expression is associated with genomic aberrations in breast cancer. *Cancer Research*.

[23] Zhu F. X., Wang X. T., Zeng H. Q., Yin Z. H., Ye Z. Z. (2019). A predicted risk score based on the expression of 16 autophagy-related genes for multiple myeloma survival. *Oncology Letters*.

[24] Liu Y., Yang N., Peng X., Liu G., Zhong H., Liu L. (2019). One-Lincrna and five-Mrna based signature for prognosis of multiple myeloma patients undergoing proteasome inhibitors therapy. *Biomedicine & Pharmacotherapy*.

[25] Prideaux S. M., Conway O'Brien E., Chevassut T. J. (2014). The genetic architecture of multiple myeloma. *Advances in Hematology*.

[26] Greipp P. R., San Miguel J., Durie B. G. (2005). International staging system for multiple myeloma. *Journal of Clinical Oncology*.

[27] Shaughnessy J., Jacobson J., Sawyer J. (2003). Continuous absence of metaphase-defined cytogenetic abnormalities, especially of chromosome 13 and hypodiploidy, ensures long-term survival in multiple myeloma treated with total therapy I: interpretation in the context of global gene expression. *Blood*.

[28] Fonseca R., Blood E., Rue M. (2003). Clinical and biologic implications of recurrent genomic aberrations in myeloma. *Blood*.

[29] Sotiriou C., Piccart M. J. (2007). Taking gene-expression profiling to the clinic: when will molecular signatures become relevant to patient care?. *Nature Reviews. Cancer*.

[30] Shaughnessy J. D., Zhan F., Burington B. E. (2006). A validated gene expression model of high-risk multiple myeloma is defined by deregulated expression of genes mapping to chromosome 1. *Blood*.

[31] Decaux O., Lode L., Magrangeas F. (2008). Prediction of survival in multiple myeloma based on gene expression profiles reveals cell cycle and chromosomal instability signatures in high-risk patients and hyperdiploid signatures in low-risk patients: a study of the intergroupe francophone du Myélome. *Journal of Clinical Oncology*.

[32] Cho S. F., Chang Y. C., Chang C. S. (2014). Malat1 long non-coding Rna is overexpressed in multiple myeloma and may serve as a marker to predict disease progression. *BMC Cancer*.

[33] Zhou M., Zhao H., Wang Z. (2015). Identification and validation of potential prognostic Lncrna biomarkers for predicting survival in patients with multiple myeloma. *Journal of Experimental & Clinical Cancer Research*.

[34] Hu A. X., Huang Z. Y., Zhang L., Shen J. (2017). Potential prognostic long non-coding Rna identification and their validation in predicting survival of patients with multiple myeloma. *Tumour Biology*.

[35] Qi L., Zhang T., Yao Y. (2019). Identification of Lncrnas associated with lung squamous cell carcinoma prognosis in the competitive endogenous Rna network. *PeerJ*.

[36] Li M., Liu Y., Zhang X., Liu J., Wang P. (2018). Transcriptomic analysis of high-throughput sequencing about Circrna, Lncrna and Mrna in bladder cancer. *Gene*.

[37] Wang Q., Zhang D., Feng W. (2019). Long noncoding Rna Tspoap1 antisense Rna 1 negatively modulates type I Ifn signaling to facilitate influenza a virus replication. *Journal of Medical Virology*.

[38] Giulietti M., Righetti A., Principato G., Piva F. (2018). Lncrna co-expression network analysis reveals novel biomarkers for pancreatic cancer. *Carcinogenesis*.

