# Supplementary figures and images for: Identification and Validation of a Potential Prognostic 7-lncRNA Signature for Predicting Survival in Patients with Multiple Myeloma
Source: Biomed Res Int. 2020 Nov 5;2020:3813546. doi: 10.1155/2020/3813546 (PMC7661128; doi:10.1155/2020/3813546)

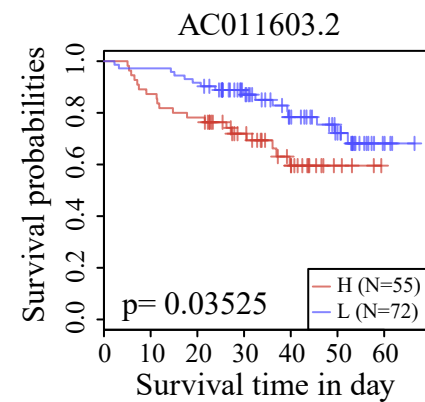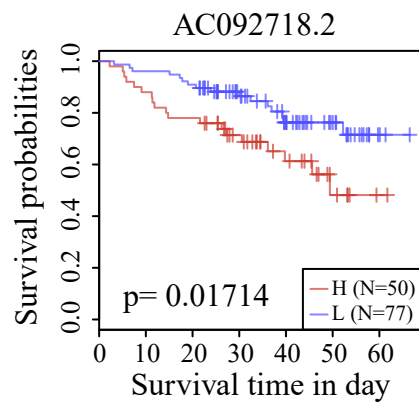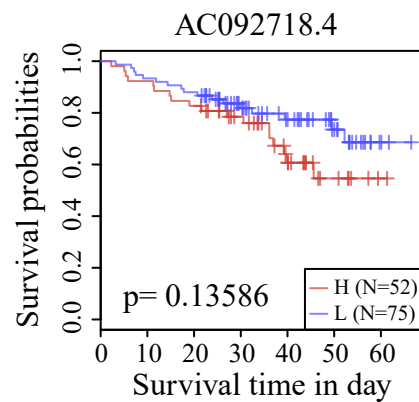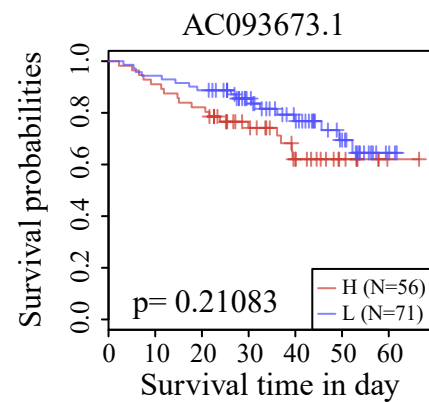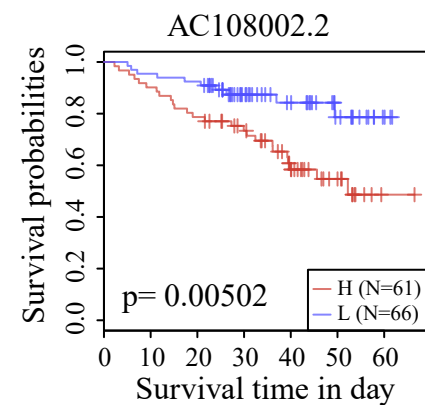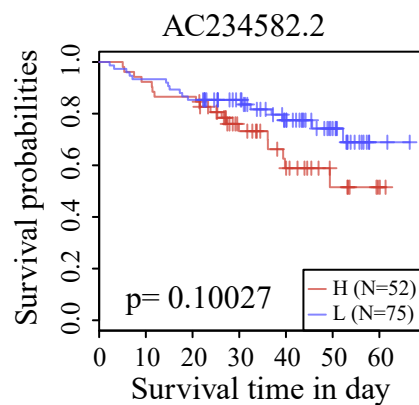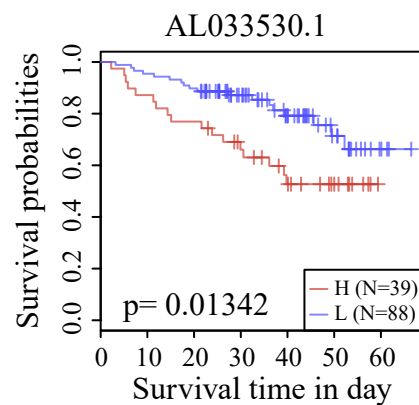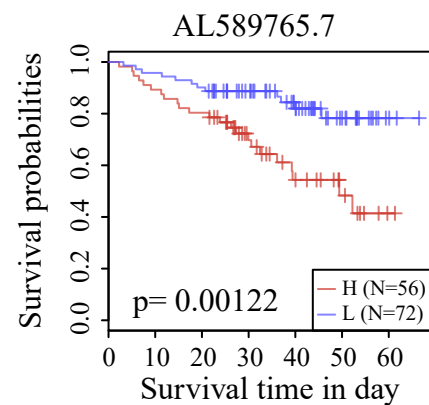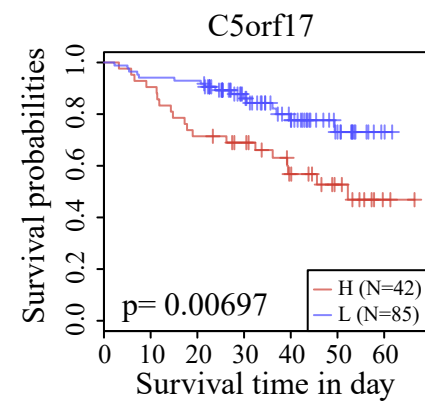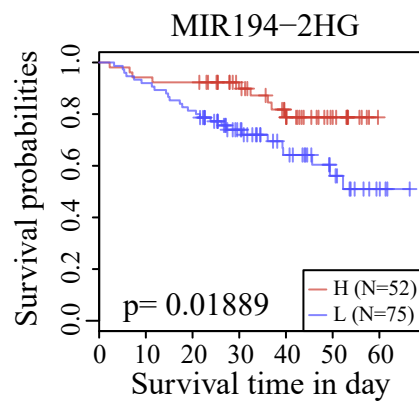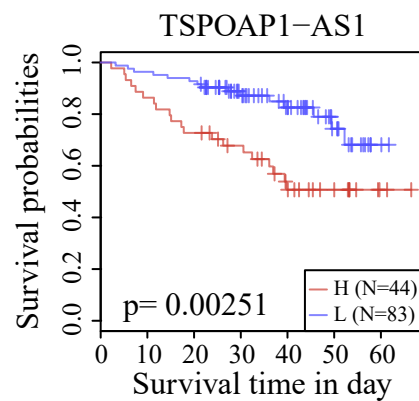

Supplement: Supplementary Materials — KM curve analysis of 11 lncRNAs. [file 3813546.f1.pdf]
